# Electrically pumped photonic integrated soliton microcomb

**DOI:** 10.1038/s41467-019-08498-2

**Published:** 2019-02-08

**Authors:** Arslan S. Raja, Andrey S. Voloshin, Hairun Guo, Sofya E. Agafonova, Junqiu Liu, Alexander S. Gorodnitskiy, Maxim Karpov, Nikolay G. Pavlov, Erwan Lucas, Ramzil R. Galiev, Artem E. Shitikov, John D. Jost, Michael L. Gorodetsky, Tobias J. Kippenberg

**Affiliations:** 1 Swiss Federal Institute of Technology Lausanne (EPFL), 1015 Lausanne, Switzerland; 2 Russian Quantum Center, Moscow, Russia 143025; 3 Moscow Institute of Physics and Technology, Dolgoprudny, Russia 141700; 4 Faculty of Physics, M.V. Lomonosov Moscow State University, Moscow, Russia 119991; 5 MicroR Systems Sarl, 1003 Lausanne, Switzerland; 6 Key Laboratory of Specialty Fiber and Optical Access Networks, Shanghai University, 200343 Shanghai, China

## Abstract

Microcombs provide a path to broad-bandwidth integrated frequency combs with low power consumption, which are compatible with wafer-scale fabrication. Yet, electrically-driven, photonic chip-based microcombs are inhibited by the required high threshold power and the frequency agility of the laser for soliton initiation. Here we demonstrate an electrically-driven soliton microcomb by coupling a III–V-material-based (indium phosphide) multiple-longitudinal-mode laser diode chip to a high-*Q* silicon nitride microresonator fabricated using the photonic Damascene process. The laser diode is self-injection locked to the microresonator, which is accompanied by the narrowing of the laser linewidth, and the simultaneous formation of dissipative Kerr solitons. By tuning the laser diode current, we observe transitions from modulation instability, breather solitons, to single-soliton states. The system operating at an electronically-detectable sub-100-GHz mode spacing requires less than 1 Watt of electrical power, can fit in a volume of ca. 1 cm^3^, and does not require on-chip filters and heaters, thus simplifying the integrated microcomb.

## Abstract (web summary)

Chip-based frequency combs promise many applications, but full integration requires the electrical pump source and the microresonator to be on the same chip. Here, the authors show such integration of a microcomb with < 100 GHz mode spacing without additional filtering cavities or on-chip heaters.

## Introduction

Optical frequency combs have revolutionized time-keeping and frequency metrology over the past two decades, and have found a wide variety of applications^1,2^. Microresonator-based Kerr frequency combs (Kerr microcombs) have provided a route to compact chip-scale optical frequency combs, with broad optical bandwidth and repetition rates in the microwave to terahertz domain (10 GHz−1 THz)^3,4^. Their compact and low-power nature could enable utilization in mobile or airborne applications beyond research laboratories, including operation in space^5^. The observation that such microcombs can be operated in the dissipative Kerr soliton (DKS) regime (soliton microcombs) has enabled fully coherent microcombs^6,7^. DKS exhibits a rich set of nonlinear optical phenomena such as soliton Cherenkov radiation (also known as dispersive waves), which can extend the spectral bandwidth of the frequency comb^8^. Soliton microcombs have been applied in counting of the cycles of light^9^, coherent communication^10^, ultrafast ranging^11,12^, dual-comb spectroscopy^13^, low-noise microwave generation^14^, and optical frequency synthesis^15^. The full photonic integration of soliton microcombs in a single, compact, and electrically driven package would allow mass-manufacturable devices compatible with emerging high-volume applications such as laser-based ranging, or sources for dense wavelength division multiplexing for data center-based optical interconnects. Via advances in silicon photonics, such level of integration has been achieved for lasers^16^, modulators^17^, and a wide range of passive and active elements, which are already commercially available. Photonic integration of soliton microcombs requires not only the integration of nonlinear high-*Q* microresonators on chip but also an on-chip solution for the narrow linewidth seed lasers with output power levels that are sufficient for soliton initiation, as well as any laser tuning mechanism used in the soliton excitation process^7,8,18,19^. On one hand, photonic integration of high-*Q* microresonators suitable for the soliton formation has advanced significantly, in particular using Si_3_N_4_—a CMOS-compatible material used as a capping layer^20^. The platform possesses several advantageous properties, including a high Kerr nonlinearity, large flexibility for dispersion engineering, outer-space compatibility^5^, and a large bandgap (~5 eV), thus free from two-photon absorption in the telecommunication band. All these advantages facilitate soliton formation in Si_3_N_4_ microresonators^8^. In a related effort, ultrahigh-*Q* SiO_2_ air-clad microresonators have recently been integrated with Si_3_N_4_ waveguides for soliton generation^21^. On the other hand, efforts to combine integrated photonic microresonators with chip-scale lasers, such as those developed in silicon photonics, have recently been made^15^. Yet, these and other approaches are still optically pumped with stand-alone bulk laser modules, and typically employ additional amplifiers for soliton initiation to overcome coupling losses and the low *Q*-factors of integrated photonic resonators. Likewise, the use of silicon photonics-based lasers is presently compounded since the threshold of soliton formation usually exceeds the laser’s output power (few-mW scale). Recent advances in fabrication of high-*Q* Si_3_N_4_ photonic integrated microresonators (intrinsic *Q*_0_ > 1 × 10^7^)^22–24^ suggest that electrically driven microcombs that employ chip-scale laser diodes—compatible with scalable manufacturing—may become viable.

Here we demonstrate an electrically driven, and current-initiated, soliton microcomb significantly simplifying photonic integration. The integrated device has a volume of ca. 1 cm^3^, and uses a commercially available semiconductor laser diode chip. This device consumes less than 1 Watt of electrical power and produces a soliton microcomb with sub-100-GHz line spacing. By using high-*Q* (*Q*_0_ > 1 × 10^7^) photonic chip-scale Si_3_N_4_ microresonators fabricated using the photonic Damascene reflow process^24,25^, in conjunction with a multiple-longitudinal-mode (multi-frequency) Fabry–Pérot InP laser diode chip, we observe self-injection locking^26,27^ in a regime where solitons are formed concurrently. Such self-injection locking with concurrent soliton formation has recently been demonstrated for bulk ultrahigh-*Q* crystalline MgF_2_ resonators^14,28^. We observe that the current tuning of the laser diode can induce transitions from the injection-locking-based single-longitudinal-mode lasing (×1000-fold reduction of linewidth) to Kerr frequency combs, breather soliton formation, followed by stable multiple and single DKS formation in the integrated microresonator. Heterodyne measurements demonstrate the low-noise nature of the generated soliton states. Such an electrically driven photonic chip-based soliton microcomb demonstrated here provides a solution for integrated, unprecedentedly compact optical comb sources suitable for high-volume applications. In comparison with a concurrent report of integrated soliton microcomb^29^, our scheme alleviates the need for on-chip Vernier filters, as well as for thermal heaters for soliton tuning^30^, which avoids extra power consumption (30 mW per heater^29^) and the complexity in both the fabrication process and the process of soliton initiation.

## Results

### Experimental setup and technique

**Fig. 1:**
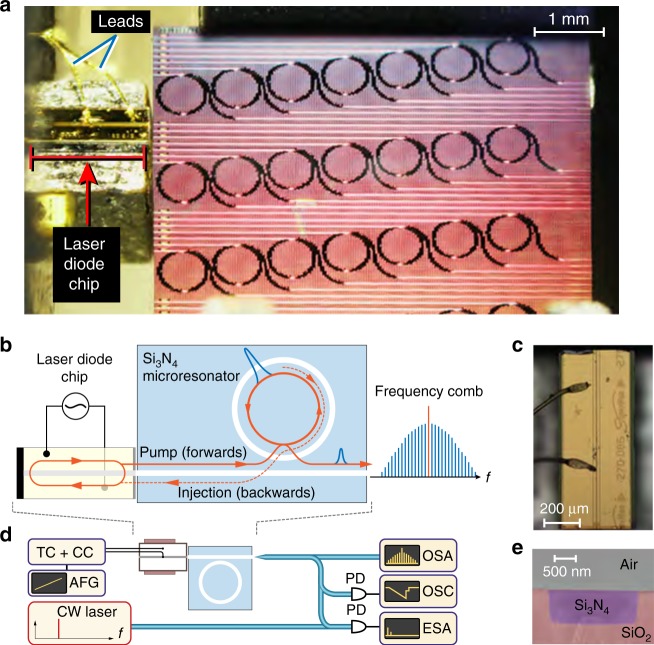
Principle of an ultra-compact, laser injection-locked soliton Kerr frequency comb. **a** Close-range photo of the experimental setup, in which the laser diode chip is butt-coupled to a Si_3_N_4_ photonic chip, which contains several microresonators. **b** Schematic representation of the laser injection-locked soliton Kerr frequency comb. An InP multi-frequency laser diode chip is directly butt-coupled to a Si_3_N_4_ photonic chip with a microresonator. **c** An optical image of the InP laser diode chip showing the magnified view. **d** Sketch of the experimental setup. The microresonator device output is characterized both in the optical domain using an optical spectral analyzer and in the radio frequency (RF) domain using an electrical-signal spectral analyzer. In addition, to assess the coherence of the frequency comb, we employ a heterodyne beatnote measurement to a selected comb tooth with a narrow linewidth reference laser. TC: temperature control module; CC: current control module; AFG: arbitrary function generator; OSA: optical spectral analyzer; OSC: oscilloscope; ESA: electrical-signal spectral analyzer. **e** False-colored scanning electron micrograph (SEM) image of the waveguide cross-section. The Si_3_N_4_ waveguide (blue) has no top SiO_2_ cladding but only side and bottom SiO_2_ cladding (red)

Figure 1 illustrates the approach taken in this work. A multi-frequency Fabry–Pérot laser diode chip (InP) is directly butt-coupled to a Si_3_N_4_ photonic chip (Fig. 1a, b). The butt-coupling scheme gives an overall insertion loss of ~6 dB (diode-chip-lensed fiber), with a double-inverse tapered structure for the light input/output coupling^31^. When the frequency of the light emitted from the laser diode coincides with a high-*Q* resonance of the Si_3_N_4_ microresonator, laser self-injection locking can take place. The process occurs due to the bulk and surface Rayleigh scattering in the microresonator, which injects a fraction of light back into the diode^27^. This provides a frequency-selective optical feedback to the laser, leading to single-frequency operation with a significant reduction of the laser linewidth.

A key step for our approach is to match the optical power requirement for soliton generation to that of the laser diode. This is achieved by employing high-*Q* Si_3_N_4_ microresonators fabricated using the photonic Damascene process, featured with a novel and critical reflow step^24^, allowing for ultra-smooth waveguide sidewalls that enable high-*Q*-factors (*Q*_0_ > 1 × 10^7^) across the entire L-band (cf. Methods).

**Fig. 2:**
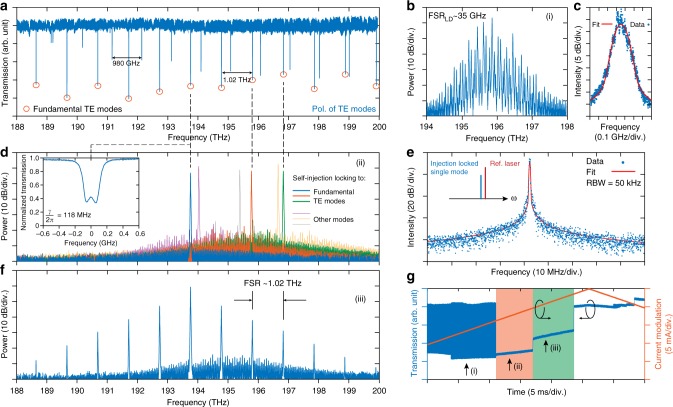
Electrically pumped soliton microcomb via laser injection-locked soliton formation. **a** Transmission spectrum of a Si_3_N_4_ microresonator of 1.02 THz free spectral range (FSR), featuring two sets of resonances: the fundamental transverse electric (TE) mode family (marked by red circles) and one high-order TE mode family. **b** The laser spectrum of the multi-frequency laser diode chip used in this experiment, corresponding to state i in **g**. **c** Measured and fitted heterodyne beat signal between the free running laser diode and a narrow linewidth reference laser (Toptica CTL1550, short-time linewidth ~10 kHz), showing 60 MHz full-width at half-maximum (FWHM) of Voigt profile. **d** (state ii in **g**): Spectra of single longitudinal mode that is injection locked to a selected resonance of the microresonator. **f** (state iii in **g**): Spectrum of the Kerr frequency comb that stems from the laser injection locking. Inset: One resonance of the fundamental TE mode showing mode splitting due to backscattering, with the estimated 118 MHz coupling strength $\left( {\frac{\gamma }{{2\pi }}} \right)$ between the forward and backward propagating modes. **e** Heterodyne beat signal between the injection-locked laser and a narrow linewidth reference laser. The measured beat signal is fitted with Voigt profile with FWHM ~186 kHz (cf. Methods). RBW: Resolution bandwidth. **g** Typical transmitted power trace measured at the chip output facet, by current modulation imposed on the laser diode, in which different states are marked: (i) noisy, multi-frequency lasing without injection locking; (ii) laser injection locking to a microresonator resonance, and simultaneous formation of low-noise single-longitudinal-mode lasing (the orange region); (iii) formation of Kerr frequency comb (the green region)

The Fabry–Pérot laser diode we employ in the experiments is centered at 1530 nm, and its emission spectrum without self-injection locking is shown in Fig. 2b. The mode spacing is 35 GHz, determined by the Fabry–Pérot cavity length. The overall maximum optical output power is ~100 mW when applying a current of ~350 mA to the diode. The electrical power consumed by the laser diode is less than 1 W. Figure 2c shows the heterodyne beatnote of the free running laser diode mode with a reference laser (Toptica CTL1550, short-time linewidth ~10 kHz), which is fitted with the Voigt profile (cf. discussions in the following and Methods), revealing both a Gaussian linewidth of 60 MHz and an estimated short-time linewidth of 2 MHz.

### Self-injection locking phenomena

We first studied self-injection locking of the laser diode chip to the photonic chip-based microresonator. This is achieved by tuning the current of the laser diode, which not only changes the optical output power but also shifts the lasing frequency via the carrier dispersion effect. Initially, the laser diode coupled to the Si_3_N_4_ chip operates multi-frequency (Fig. 2b), a regime where none of the high-*Q* microresonator modes is frequency-matched with the multi-mode laser emission from the diode. By shifting the lasing frequency of the diode via current tuning, we observe that the initially multi-frequency emission spectrum switches to single mode operation, indicative of self-injection locking. Figure 2d demonstrates that the lasing frequency coincides with a selected resonance of the microresonator, and we also observe injection locking occurring for several resonances^32^. We note that all resonances, which give rise to the laser self-injection locking, feature mode splitting as a result of backscattering (cf. the inset in Fig. 2d). The back-coupling rate for the measured resonance, extracted from its mode-splitting profile, is *γ*/2*π* = 118 MHz (cf. Methods). The presence of this back-coupling leads to an amplitude reflection coefficient (*r*) from the passive microresonator on resonance:

1 $$r \approx \frac{{2\eta {\mathrm{\Gamma }}}}{{1 + {\mathrm{\Gamma }}^2}},$$

where *η* = *κ*_ex_/*κ* characterizes coupling efficiency (*κ* = *κ*_0_ + *κ*_ex_, with *η* = 1/2 corresponding to critical coupling, and *η* ≈ 1 corresponding to strong overcoupling), and Γ = *γ*/*κ* is the normalized mode-coupling parameter that describes the visibility of the resonance split. According to ref. ^33^ this reflection can initiate self-injection locking, and give rise to a narrow linewidth of:

2 $$\delta \omega \approx \delta \omega _{{\mathrm{free}}}\frac{{Q_{{\mathrm{LD}}}^2}}{{Q^2}}\frac{1}{{16r^2(1 + \alpha _g^2)}},$$

where *Q* = *ω*/*κ* is the microresonator quality factor, *ω*/2*π* is the light frequency, and *δω*_free_/2*π* is the linewidth of the free running laser. The phase-amplitude coupling factor *α*_*g*_ is the linewidth enhancement factor, given by the ratio of the variation of the real refractive index to the imaginary refractive index of the laser diode-active region in response to a carrier density fluctuation^34^ and takes typical values from 1.6 to 7. The InGaAsP/InP multiple quantum well laser diode has *α*_*g*_ = 2.5. The laser diode quality factor *Q*_LD_ can be estimated as $Q_{{\mathrm{LD}}} \approx \frac{{\omega \tau _{\mathrm{d}}R_{\mathrm{o}}}}{{1 - R_{\mathrm{o}}^2}},$ where *R*_o_ is the amplitude reflection coefficient of the output laser mirrors and *τ*_d_ is the laser cavity round trip. The reflection coefficient is a parameter of the laser diode and is given by the laser diode manufacturer as $R_{\mathrm{o}} = \sqrt {0.05}$ as well as *α*_*g*_ = 2.5. Other experimentally determined parameters are *κ/*2*π* ≈ 110 MHz*, γ/*2*π* ≈ 118 MHz*, η* ≈ 0.64, Γ ≈ 1, and *τ*_d_ = 1/FSR_diode_ = 1/(35 GHz) = 28.6ps. The theoretical estimation for the narrowed linewidth is *δω/*2*π*~0.1 kHz. We next compare these theoretical estimates of the self-injectioned locked linewidth to experiments. The linewidth of the self-injection-locked single-longitudinal-mode laser is measured by a heterodyne measurement (see Fig. 2e). The lineshape is fitted with a Voigt profile, which represents a convolution of Lorentzian and Gaussian lineshape (cf. Methods), yielding a Gaussian contribution to the linewidth of 186 kHz. The estimated Lorentzian contribution amounts to 0.7 kHz, describing the wings of the measured beatnote. Self-injection locking leads to a narrowing of the white noise of the laser diode (Eq. 2)^33^. Therefore, this value should be compared with the Lorentzian contribution in the Voigt profile (i.e., 0.7 kHz) corresponding to a more than 1000-fold reduction in the linewidth.

Injection locking occurs also in the case where the laser cavity and microresonator are detuned from each other due to “injection pulling,” and as outlined below, is imperative to generate DKS using self-injection locking. Injection pulling is a result of a slight phase difference between the laser emission and its feedback, leading to imperfect locking^33^. The locking range is defined as the frequency range over which the laser diode emission self-injection locks to the high-*Q* microresonator resonance and follows the expression^33^:

3 $${\mathrm{\Delta }}\omega _{{\mathrm{lock}}} \approx r\sqrt {1 + \alpha _g^2} \frac{\omega }{{Q_{{\mathrm{LD}}}}}.$$

The theoretically estimated locking range exceeds Δ*ω*_lock_/2*π* ≈ 30 GHz.

### Kerr frequency comb generation via self-injection locking

Experimentally, we can access injection pulling by tuning the current of the laser diode, allowing the laser frequency to be changed concurrently with the self-injection locking, providing thereby a frequency scan over the resonance—a prerequisite for DKS formation^7^. Figure 2g shows the optical output power (transmission) trace as a function of the current tuning, where self-injection locking is deterministically observed. An initial chaotic power trace (state (i) in Fig. 2g) is switched to a step-like pattern (state (ii) in Fig. 2g, the orange marked region). The average output power reduces during the switching since the self-injection leads to single-longitudinal-mode operation, with enhanced power being coupled into the high-*Q* resonance of the Si_3_N_4_ microresonator. Most significantly, upon further tuning the current, a second step-like pattern in the power trace is observed (state (iii) in Fig. 2g, the green marked region), corresponding to the formation of a (low noise) Kerr frequency comb. Indeed, at high optical power levels (typically setting the current to be ~300 mA), Kerr comb generation was observed upon tuning the current, as shown in Fig. 2f. This phenomenon relies critically on the *Q*-factor of the Si_3_N_4_ microresonator, allowing sub-mW threshold power for parametric oscillations (cf. Methods).

### DKS with an electronically detectable mode spacing

**Fig. 3:**
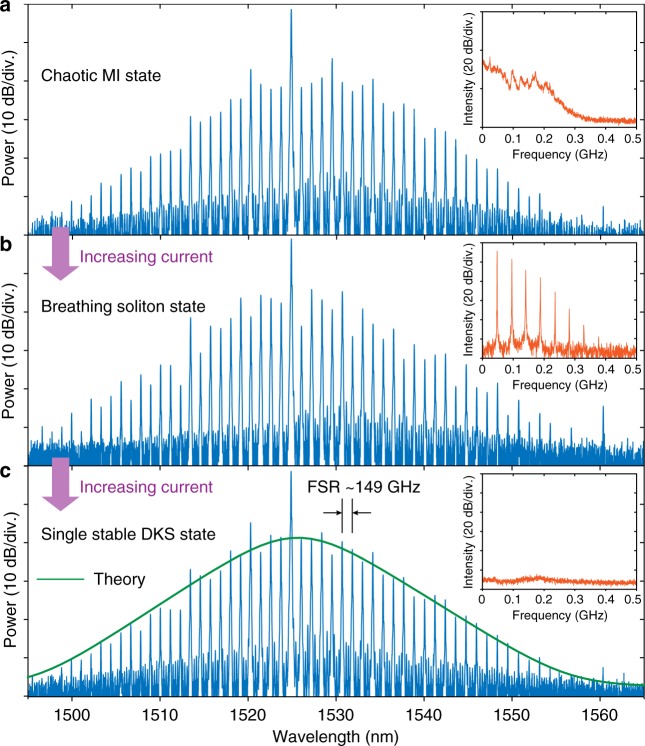
Soliton comb generation with self-injection locking. Evolution of Kerr frequency comb in the regime of laser self-injection locking, from noisy state in the operation regime of modulation instability (MI) (**a**) to breathing state (**b**), and eventually to a low-noise state (**c**) showing the formation of a dissipative Kerr soliton (DKS) in the microresonator, where the spectrum is a hyperbolic secant envelope (green-solid line showing the fitting of the spectral envelope). Each inset shows the low-frequency radio frequency (RF) spectrum corresponding to each state. The current imposed to the diode is initially set ~300 mA and the increase to evoke the transitions is within 1 mA. The Si_3_N_4_ microresonator in this measurement has a free spectral range (FSR) of 149 GHz

**Fig. 4:**
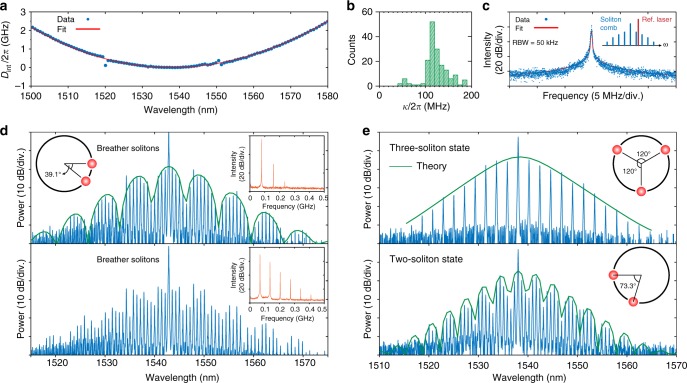
Laser injection-locked multiple breathing and dissipative Kerr solitons. **a** Measured and fitted dispersion landscape in a Si_3_N_4_ microresonator (cross-section 1.58 × 0.75 μm^2^) (cf. Methods), which has the FSR = 92.4 GHz, and the second-order dispersion element indicating the anomalous group velocity, *D*_2_/2*π* ≈ 1.56 MHz. **b** Histogram of resonance linewidths that are ~110 MHz, corresponding to a loaded *Q*-factor ~1.8 × 10^6^. **c** Heterodyne beat signal between the sideband of soliton Kerr frequency comb and the narrow linewidth reference laser. The measured beat signal is fitted with Voigt profile with full-width at half-maximum (FWHM) ~201 kHz (cf. Methods). RBW: Resolution bandwidth. **d**, **e** Showcase of multiple dissipative solitons formed in Si_3_N_4_ microresonators, in the breathing state (**d**) as well as in the low-noise stable soliton state (**e**), the fitting of the spectrum envelope (green-solid lines) further shows the relative position of solitons circulating in the micro-ring cavity (schematic insets). The low-frequency RF spectra corresponding to breather solitons are also shown as insets. Spectra in **d** and **e** are generated in Si_3_N_4_ microresonators with a free spectral range (FSR) of ~88 and ~92 GHz, respectively

We next investigated if self-injection locking can also be observed in devices with an electronically detectable mode spacing (149 and <100 GHz), and critically if it can also enable operation in a regime where DKS are formed concurrently. Figure 3a shows the self-injection-locked Kerr comb generation in a microresonator with a free spectral range (FSR) of 149 GHz. Significantly, not only were Kerr combs observed but also switching into the DKS regime^7^. Upon self-injection locking, and via current tuning we first excite a Kerr comb in a low-coherence state, as evidenced by the noise in the low-frequency radio frequency (RF) spectrum (inset in Fig. 3a). We emphasize that for such low repetition rates the amplitude noise is still a valid indicator of the frequency comb’s coherence, in contrast to terahertz mode spacing resonators where the noise can be located at high RF frequencies (>1 GHz)^35^. Importantly, we observe, that upon increasing the current to the diode further (${\cal O}$(mA)), which leads to a laser detuning increase by injection pulling, the low-coherence comb state is turned into an intermediate oscillatory state. That can be identified as a breather DKS (Fig. 3b)^36^, where the soliton exhibits periodic oscillations both in the power and in the pulse duration. The RF spectrum shows the breathing frequency at ~490 MHz exhibiting harmonics, see inset in Fig. 3b. Such soliton breathing dynamics (breather solitons) have been studied previously^36^, and in particular the feature that breathing frequency depends on the laser detuning  is also observed in the present work via the current tuning scheme. The observation of a DKS breathing state demonstrates that the injection pulling enables operation in the effectively red detuned regime, required for soliton generation. Further increasing the laser current, we observe a transition to a low-noise comb state, demonstrating the formation of stable DKS as shown in Fig. 3c. The spectral envelope of the frequency comb exhibits a secant-squared profile, corresponding to a single soliton circulating in the resonator, with the breathing oscillations absent from the RF spectrum (inset in Fig. 3c). This transition, which we induce here by current tuning only, has been achieved in previous work by tuning the laser over the resonance from the blue to the effectively red detuned side^7^. Most significantly, to corroborate operation in the soliton state we verify the coherence via a heterodyne beatnote measurement^2^. The heterodyne beatnote of a soliton comb tooth with a narrow linewidth reference laser is shown in Fig. 4c. The measured heterodyne beatnote linewidth is comparable to that of the injection-locked laser (cf. Figure 2e), that is, the Gaussian linewidth is 201 kHz and the estimated short-time Lorentzian linewidth (that describes the wings of the beatnote only) is 1 kHz. These values indicate no degradation of the coherence during the process of soliton comb generation via laser self-injection locking.

DKS formation via laser self-injection locking was also observed in Si_3_N_4_ microresonators with FSRs below 100 GHz, an electronically detectable repetition rate, where due to the high *Q*-factors (*Q*_0_ ~ 8 × 10^6^) enabled by the photonic Damascene reflow process, soliton combs could still be generated^25^. Figure 4a, b show a dispersion measurement of the microresonator (cf. Methods), where the FSR is read as 92.4 GHz. The parabolic dispersion profile shows quadratic contribution from an anomalous group velocity dispersion (GVD) to be: *D*_2_/2*π* ≈ 1.56 MHz, centered at a wavelength ~1540 nm. The loaded resonance linewidth *κ*/2*π* is ca. 110 MHz (Fig. 4b), corresponding to an over-coupled regime of the microresonator (the intrinsic loss rate is *κ*_0_/2*π* < 30 MHz). In these type of microresonators, multiple dissipative solitons are observed, shown in Fig. 4d, e, not only in the breathing state but in the low-noise stable soliton state as well. The spectral envelope reveals a multi-soliton state as a result of interfering Fourier components of the solitons. By fitting these spectral envelopes (cf. Methods), we can resolve the number of solitons and estimate their relative positions, illustrated as insets in Fig. 4d, e. The overall transmitted optical power, consisting of both the comb power and the residual pump power, is measured ~11 mW (cf. Methods).

## Discussion

In summary, we have demonstrated a route to an ultra-compact, cost-effective soliton frequency comb in photonic integrated Si_3_N_4_ microresonators, via laser self-injection-locking with off-the-shelf laser diodes. Self-injection-locking leads to a significant stabilization of a laser diode, eliminates the need for complicated soliton generation methods. We observed power-efficient soliton combs in microresonators with different FSRs, particularly for FSR below 100 GHz. This approach offers a dramatic reduction in size, cost, and weight, and also offers simplified heterogeneous integration, in particular as no wafer bonding is required unlike for silicon photonic III–V lasers. This approach provides a route to scalable manufacturing of soliton microcombs for future high-volume applications.

## Methods

### Fabrication and characterization of Si_3_**N**_**4**_ microresonator chip

The photonic integrated Si_3_N_4_ chips are fabricated using the photonic Damascene reflow process. Waveguide and resonator patterns were defined by deep-ultraviolet (DUV) stepper lithography and transferred to the SiO_2_ preform via dry etching. A preform reflow step was used to reduce the waveguide sidewall roughness caused by dry etching^24,37^, allowing for ultra-smooth waveguides and leading to high-*Q*-factors for the microresonators. Optimized chemical mechanical polishing (CMP) allows precise control of the waveguide height to 750 ± 20 nm, measured over the full 4-in. wafer scale. No top cladding was deposited onto the Si_3_N_4_ waveguides. The precise dimension control by both the lithography (mainly in the waveguide width) and CMP (in the height) enables samples of the same design to have the identical geometry at different positions on the wafer.

The microresonator is coupled to the bus waveguide on the chip through the evanescent field. Light is coupled onto the Si_3_N_4_ chip via double inverse nanotapers^31^ on the bus waveguides at both the input and output facets, that is, from the laser diode chip to the microresonator chip and from the microresonator chip to a lensed fiber which collects the comb spectrum. In addition, the bus waveguide’s geometry is designed to achieve a high coupling ideality with reduced parasitic losses^38^.

The microresonator dispersion can be extracted by measuring the transmission spectrum, which is calibrated by a standard optical frequency comb^39,40^. The dispersion of the microresonator is represented in terms of resonant frequency deviation with respect to a linear grid, namely:

4 $$D_{{\mathrm{int}}} = \omega _\mu - (\omega _0 + \mu D_1) = \mathop {\sum}\limits_{m \ge 2} \frac{{\mu ^mD_m}}{{m!}},$$

where *ω*_*μ*_ is the physical resonant frequencies of the microresonator. A central resonance (to which the laser is injection locked) is given the index *μ* = 0. *D*_1_ = 2*π* × FSR is the repetition frequency. The second-order element *D*_2_ is the GVD of the microresonator and *D*_2_ > 0 represents the anomalous GVD. When the dispersion is described to the second order, the dissipative and nonlinear optical resonator can be described by the Lugiato–Lefever equation^41^, which is equivalent to the coupled mode equation. Each resonance is fitted using the model based on coupled mode theory^42,43^ from the transmission spectrum. The resonance linewidth reflects the total loss rate (*κ*) of the microresonator, which consists of both the intrinsic loss rate (*κ*_0_) and the external coupling rate *κ*_ex_, that is, *κ* = *κ*_0_ + *κ*_ex_. To extract the intrinsic *Q*-factor (*Q*_0_), highly under-coupled microresonators are measured, that is, *κ*_ex_ → 0.

In this work, there are three sets of Si_3_N_4_ microresonators in terms of different FSRs: ~1 THz, ~150 GHz, and <100 GHz. The microresonator corresponding to results shown in Fig. 2f has: *Q*_0_ ≈ 6 × 10^6^, FSR = 1.02 THz, *D*_2_/2*π* ≈ 188 MHz, for fundamental TE mode. The microresonator width is 1.53 μm. The microresonator corresponding to results shown in Fig. 3 has: *Q*_0_ ≈ 6.5 × 10^6^, FSR = 149 GHz, *D*_2_/2*π* ≈ 3.90 MHz (fundamental TE mode), the microresonatore width is 1.58 μm. The microresonators corresponding to results shown in Fig. 4 have: *Q*_0_ ≈ 8.2 × 10^6^; (for Fig. 4d) FSR = 88.6 GHz, *D*_2_/2*π* ≈ 1.10 MHz (fundamental TE mode), the microresonator width is 1.58 μm; (for Fig. 4e) FSR = 92.4 GHz, *D*_2_/2*π* ≈ 1.56 MHz (fundamental TE mode), the microresonator width is 1.58 μm.

Such high *Q*-factors have already enabled direct soliton comb generation in microresonators without amplification of the seed laser^25^. The threshold power for parametric oscillation can be as low as sub-milli-Watt (critical coupled), which is calculated as:

5 $$P_{{\mathrm{th}}} = \frac{{\kappa ^2n^2V_{{\mathrm{eff}}}}}{{4\omega cn_2}},$$

where *n* is the refractive index, *V*_eff_ indicates the effective modal volume, *ω* is the angular frequency of light, *c* the speed of light in vacuum, and *n*_2_ is the nonlinear refractive index. For Si_3_N_4_ microresonators with FSR ~1 THz, we have *n*  ≈1.9, *V*_eff_  ≈1.5 × 10^−16^ μm^3^, and *n*_2_ ≈2.4 × 10^−19^ m^2^/W. Hence, the threshold power is as low as *P*_th_ ≈ 0.62 mW.

### DKS comb power

Multiple DKS in the microresonator with FSR ~92.4 GHz are generated when applying a current ~280 mA to the diode chip, corresponding to an optical output power of ~50 mW. The output power is measured as ~11 mW, collected by using a lensed fiber at the output chip facet, indicating a coupling efficiency of ~22% (overall insertion loss −6.6 dB). The optical power in the bus waveguide is estimated to be ~23.5 mW, which has been demonstrated to be sufficient to excite DKS in high-*Q* Si_3_N_4_ microresonators^25^.

### Heterodyne beat signal and fitting function

The heterodyne measurement is used to assess the coherence of the generated soliton comb, as its lineshape reveals the frequency noise spectral density with respect to the reference laser. In fact, the frequency noise may consist of both the white noise (resulting in a Lorenztian lineshape) and the flicker noise (corresponding to a Gaussian lineshape). Therefore, we employ the Voigt profile^44^ to fit the beat signal, which represents the convolution of the Lorenztian (*L*(*f*)) and the Gaussian (*G*(*f*)) lineshapes, that is:

6 $$V(f) = {\int}_{ - \infty }^{ + \infty } G(f\prime ;\sigma )L(f - f\prime ;,\psi ){\mathrm{d}}f\prime ,$$

7 $$G(f;\sigma ) = \frac{{{\mathrm{exp}}^{ - f^2/2\sigma ^2}}}{{\sigma \sqrt {2\pi } }},$$

8 $$L(f;\psi ) = \frac{\gamma }{{\pi (f^2 + \psi ^2)}},$$

where *f* indicates the frequency shift with respect to the center of the beat signal, in the radio frequency domain, and *σ* and *ψ* scale the linewidth. To initiate the fitting we assume that, on the wings of the beat profile, the signal is mostly contributed by the white noise that determines the intantaneous linewidth described by *ψ*. In contrast, around the center of the beat profile, the signal is also contributed by flicker noise depending on, for example, the acquisition time of the electrical-signal spectral analyzer (ESA), as well as the stability of current or temperature controller. This part of noise is scaled by *σ*. The full-width at half-maximum (FWHM) of the Gaussian lineshape is then Δ*f*_G_ = 2*σ* and Δ*f*_L_ = 2*ψ* for the Lorentzian.

### DKS comb spectral fitting

It is known that *N* identical solitons circulating in the resonator produce a spectral interference on the single soliton spectrum:^7,8^

9 $$S^{(N)}(\mu ) = S^{(1)}(\mu )\left( {N + 2\mathop {\sum}\limits_{j \ne l} {\mathrm{cos}}(\mu (\phi _j - \phi _l))} \right).$$

Here *ϕ*_*i*_ ∈ [0, 2*π*] is the position of the *i*th pulse along the cavity round trip, *μ* is the comb mode index relative to the pump laser frequency and *S*^(1)^(*μ*) is the spectral envelope of a single soliton following an approximate secant hyperbolic squared:

10 $$S^{(1)} \approx A\,{\mathrm{sech}}^2\left( {\frac{{\mu - \mu _{\mathrm{c}}}}{{{\mathrm{\Delta }}\mu }}} \right),$$

where *A* is the power of the comb lines near the pump, Δ*μ* is the spectral width of the comb (in unit of comb lines) and *μ*_c_ is the central mode of the soliton (to account for soliton recoil or self-frequency shift). Knowing the comb repetition rate *f*_r_, the spectral width (or pulse duration) can be retrieved: Δ*f* = *f*_r_Δ*μ*.

The spectral envelope of the single or multiple soliton states are fitted using the following procedure: first, the peaks $\tilde S(\mu )$ constituting the frequency comb are detected and labeled with their relative mode index from the pump *μ*, and the pump mode is rejected. The number of solitons *N* is estimated by taking the inverse Fourier transform of this spectrum, which yields the autocorrelation of the intracavity waveform, and detecting its peaks^8^. The set of fitting parameters {*A*, Δ*μ*, *μ*_c_, *ϕ*_*i*_|*i* ∈ [2, *N*]} is defined accordingly (the position of one soliton is arbitraly set to zero) and the expression (9) is fitted to the experimental points $\tilde S(\mu )$. When *N* solitons are perfectly equi-spaced, the repetition is multiplied by *N* and the single soliton expression can be fitted on every *N* line.

## Supplementary information

Peer Review File

## Data Availability

The code and data used to produce the plots within this paper are available at 10.5281/zenodo.2203625. All other data used in this study are available from the corresponding authors upon reasonable request.
