# Author Correction: Electrically pumped photonic integrated soliton microcomb

**DOI:** 10.1038/s41467-019-09529-8

**Published:** 2019-04-03

**Authors:** Arslan S. Raja, Andrey S. Voloshin, Hairun Guo, Sofya E. Agafonova, Junqiu Liu, Alexander S. Gorodnitskiy, Maxim Karpov, Nikolay G. Pavlov, Erwan Lucas, Ramzil R. Galiev, Artem E. Shitikov, John D. Jost, Michael L. Gorodetsky, Tobias J. Kippenberg

**Affiliations:** 1 Swiss Federal Institute of Technology Lausanne (EPFL), 1015 Lausanne, Switzerland; 2 Russian Quantum Center, Moscow, Russia 143025; 3 Moscow Institute of Physics and Technology, Dolgoprudny, Russia 141700; 4 Faculty of Physics, M.V. Lomonosov Moscow State University, Moscow, Russia 119991; 5 MicroR Systems Sarl, 1003 Lausanne, Switzerland; 6 Key Laboratory of Specialty Fiber and Optical Access Networks, Shanghai University, 200343 Shanghai, China

Correction to: *Nature Communications* 10.1038/s41467-019-08498-2, published online 08 February 2018

The original version of this Article contained an error in the first sentence of the Acknowledgements, which incorrectly read ‘This publication was supported by Contract HR0011-15-C-0055 (DODOS) from the Defense Advanced Research Projects Agency (DARPA), Defense Sciences Office (DSO).’ The correct version states ‘Microsystems Technology Office (MTO)’ in place of ‘Defense Sciences Office (DSO)’. This has been corrected in both the PDF and HTML versions of the Article.

