# Supplementary material for: Electrically pumped photonic integrated soliton microcomb
Source: Nat Commun. 2019 Feb 8;10:680. doi: 10.1038/s41467-019-08498-2 (PMC6368592; doi:10.1038/s41467-019-08498-2)
Supplement: Supplementary file 1 — Peer Review File [file 41467_2019_8498_MOESM1_ESM.pdf]

This manuscript has been previously reviewed at another journal that is not operating a transparent peer review scheme. This document only contains reviewer comments and rebuttal letters for versions considered at Nature Communications.
